# Valorization of mustard and sesame oilseed cakes for food application through eco‐innovative technologies

**DOI:** 10.1002/fsn3.3214

**Published:** 2023-01-12

**Authors:** Ifrah Usman, Ali Imran, Muhammad Umair Arshad, Farhan Saeed, Muhammad Afzaal, Saima Sana, Fakhar Islam, Ayesha Siddiqua, Usman Naeem, Saiful Islam

**Affiliations:** ^1^ Department of Food Sciences Government College University Faisalabad Pakistan; ^2^ Department of Biochemistry Government College University Faisalabad Pakistan; ^3^ University Institute of Diet & Nutritional Sciences University of Lahore Lahore Pakistan; ^4^ Institution of Nutrition and Food Science University of Dhaka Dhaka Bangladesh

**Keywords:** bioactive compounds, extraction, mustard, polyphenol, sesame

## Abstract

Agricultural waste valorization is currently getting attention across the world owing to its environmental impact and rich phytochemistry. The mandate of the current investigation was the extraction and characterization of bioactive moieties from the mustard oilseed cake/meal MOC and sesame oilseed cake/meal SOC through ultrasound extraction (UE) techniques due to its higher yield and less burden on the environment as compared to conventional extraction (CE). Purposely, the MOC and SOC were initially subjected to compositional analysis. Thereafter, bioactive moieties were extracted by using different solvents, that is, ethanol and distilled water, and by applying conventional and ultrasonic extraction techniques. The outcomes indicated that among the techniques, ultrasound exhibited the highest results, and in solvents, ethanol performed better. The treatment extracted with ethanol with UE at 10 min showed the best result for total phenolic contents (TPC) as (6.07 ± 0.03 09 g GAE/100 g MOC) and (7.09 ± 0.04 g GAE/100 g SOC), DPPH radical scavenging activity (67.3 ± 1.9 TE/100 g MOC) & (72.68 ± 1.9 TE/100 g SOC), and FRAP was recorded as (2.83 ± 0.02 g TE/100 g MOC) & (3.56 ± 0.03 g TE/100 g SOC). The higher antioxidant potential showed that the mustard and sesame waste holds significant therapeutic potential owing to its rich antioxidant profile and thus should be utilized for the development of functional products against lifestyle‐related disorders. In conclusion, ultrasound is a better technique for maximum as well as accurate extraction, with ethanol exhibiting as a better solvent for this process with more yields as compared to distilled water.

## Graphical abstract

Value addition of agriculture waste.

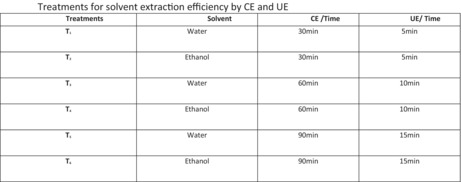

## 1 INTRODUCTION

Food security is defined as reliable social, economic, and physical access to safe and sufficient amounts of food. It is estimated that in 2020, about 12% of the total population was food insecure accounting for almost 928 million people. In Pakistan, the situation is also very alarming, as 60% of the total population's food is secure. The food waste or byproducts may be from agriculture production, inappropriate postharvest handling or storage, and negligible processing or packaging during distribution or consumption. The ever‐increasing volume of byproducts or waste from agro‐based industries has turned out to be a major trouble for industrialists (Sadh et al., 2018). Mostly, the agro waste or byproducts are untreated, unutilized, and disposed of in inappropriate methods like burning, land, filling, dumping, etc., which result in harmful environmental impact (Cecilia et al., 2019). The utilization of agricultural waste or byproducts requires less waste disposal, which is more profitable, and more economical. Commonly, agro‐industrial wastes include two types, that is*,* waste from agriculture and waste from industry. Waste from agriculture includes field waste and process waste. Field wastes or residues include seeds, pots, stems, leaves, and stalks, while the process residues are obtained as a result of crop processing including peel, roots, tubers, stubble, etc., which can be utilized for animal feed. The productions in food industries such as beverages, confectionary, bakeries, and oil generate a large volume of food waste or byproducts that accounts for food industrial waste. Oilseeds are generally known as the most common source of ingredients used in functional foods. Oilseeds are loaded with fiber, fats, vitamins, proteins, carbohydrates, phytochemicals (such as phenolic compounds, tocopherols, flavonoids, tocotrienols, and lignans) and a few antinutritional factors (e.g., phytates glucosinolates) (Ramachandran et al., 2007). Oilseeds, because of their antioxidant potential, help to combat different diseases, especially different types of cancer (Abiodun, 2017). Oil meals or oil cakes are byproducts that remain after the extraction of oil from oil seeds. There are two types of oil cakes: edible oil cakes and non‐edible oil cakes. The nutritional value of edible oil cakes is higher than that of non‐edible oil cakes. The protein content of edible oil cakes varies from 15% to 50% (www.seaoWndia.com). Fruits, vegetables, and cereals are among the top food groups which generate maximum waste or byproducts followed by other groups such as dairy, meat, and eggs. On average, globally, approx. 65 kg per year of food is wasted by each person (25% wasted vegetables, 24% wasted cereals, and 12% wasted fruits) (Chen et al., 2020). *Brassicaceae* oilseed crops such as *Brassica juncea* (mustard) are important due to their ability to support the ever‐growing biofuel industry. The extracts of mustard (Brassicaceae seed metals) have tremendous antioxidant activity resulting from the phenolic compounds present in them (Shahidi et al., 1994). *Sesame indicum* belongs to the Pedaliaceae family. Sesame seeds are vital oilseed crops. Sesame seeds have various phenolic compounds such as ferulic, cinnamic coumaric, and vanillic acids. Mohdaly et al. (2013, 2013) report showed significant biological properties. It was confirmed by various in vitro studies that phenolics and lignans existing in sesame seeds showed excellent antioxidant properties (Suja et al., 2004; Kumar & Singh, 2015; Sarkis et al. 2014). Extraction is the method that is used to obtain extracts. By employing different techniques like soxhlet, microwave‐assisted extraction, orbital shaker, supercritical fluid extraction, and ultrasound‐assisted extraction, antioxidants have been isolated from different plants (Sultana et al., 2009). For the selection of the optimal technique for antioxidant extraction, the choice of solvent is the most critical. Mostly organic solvents are used for this purpose (Kaneria et al., 2012). Conventional extraction techniques include maceration, digestion, infusion and Soxhlet extraction, etc. Extraction by these methods has been done for many years. Soxhlet and maceration are the most commonly used techniques. Ultrasound technique, by increasing mass transfer and bursting cellular matrix, releases a maximum amount of targeted compounds. In this method, as a result of exposure to ultrasound, acoustic cavitation activity results in microbubbles formation and grows in the liquid phase. Due to pressure changes, it oscillates swiftly. In case of plant material, ultasound extraction (UE) waves modify the cavitation effect as well as physiochemical properties to stimulate the extractable compounds' release and enhance mass transport by cell wall disruption. The aim of current investigation was to evaluate extraction efficiencies of oilseed cakes (mustard and sesame) from conventional and ultrasonic techniques using distilled water and ethanol as solvents.

## 2 MATERIALS AND METHODS

### 2.1 Procurement of raw material

Oilseed meal/cake generated from mustard and sesame seeds during processing was collected from the local market of Faisalabad. The oilseed cakes were cleaned to remove dirt, dust, and foreign materials, and were put in polythene bags and then stored in a refrigerator until used. After that, the extract was prepared by using solvents, distilled water, and 70% ethanol.

### 2.2 Compositional analysis

#### 2.2.1 Proximate analysis

Proximate composition of mustard and sesame oilseed cake/meals was calculated through the determination of moisture, fat, protein, fiber, and ash by following the guidelines of Kaur et al. (2014), whereas nitrogen free extract (NFE) was calculated using the subtraction method (AACC, 2000).

#### 2.2.2 Mineral analysis

In mineral analysis, the mustard and sesame oilseed cake/meal samples were determined for minerals like sodium (Na), potassium (K), calcium (Ca), magnesium (Mg), copper (Cu), and phosphorus (P) by the standard protocol of Costa et al., (2002). The first two minerals were estimated by using a Flame Photometer‐410 (Sherwood Scientific Ltd., Cambridge), while the remaining were estimated by using an atomic absorption spectrophotometer (Varian AA240, Australia).

### 2.3 Preparation of antioxidant extract

The extraction of phenolics from mustard and sesame oilseed meal was carried out through the Soxhlet extraction method. For the preparation of extracts, that is, ethanol, distilled water solvents were used. Samples were placed in the respective apparatus at room temperature for 7 h. From every flask, the supernatant was filtered using a muslin cloth. From the supernatant, the solvent was separated in a rotary vacuum at 50°C and the crude extract was left behind. (Eyela, N‐N series, Japan). Each sample extract was weighed to evaluate the antioxidant extract yield and storage was done at 4°C.

### 2.4 Analysis of antioxidant extract

The extract of antioxidants gained from mustard and sesame oilseed cake was evaluated for their antioxidant potential by various parameters such as TPC (total phenolic contents), DPPH (1,1‐diphenyl‐2‐picrylhydrazyl) assay, and FRAP (ferric‐reducing antioxidant power) assay (Table 1).

**TABLE 1:** Treatments for solvent extraction efficiency by CE and UE

Treatments	Solvent	CE/time	UE/time
T_1_	Water	30 min	5 min
T_2_	Ethanol	30 min	5 min
T_3_	Water	60 min	10 min
T_4_	Ethanol	60 min	10 min
T_5_	Water	90 min	15 min
T_6_	Ethanol	90 min	15 min

Treatment is described in Table 1.

### 2.5 Extraction procedures

#### 2.5.1 Conventional extraction

Extraction from mustard and sesame oilseed cake was done separately following Rodriguez‐Saona and Wrolstad (2001) with slight modifications. Five grams of sample (mustard and sesame oilseed cake) was added in 100 ml of water and ethanol solvent at an interval of 30, 60, and 90 min at 60°C temperature. By using a muslin cloth, these extracts were filtered. Then, these extracts were stored in air‐tight bottles. These extracts were kept at refrigeration temperature to prevent fungal attack. These extracts were stored in air‐tight bottles.

#### 2.5.2 Ultrasound‐assisted extraction

The ultrasonic extraction of bioactive moieties was estimated by following the procedures of Rusak et al. (2008) with slight modifications. As extraction solvents, ethanol and distilled water were utilized. Three different parameters were as follows:

Temperature (30°C, 40°C, and 50°C), time (30, 60, and 90 min CE) (5, 10, and 15 min US), sample to the solvent ratio (10:50), and amplitude (20%, 30%, and 40%).

#### 2.5.3 Poly‐phenols extraction

Bioactive polyphenols from the mustard and sesame oilseed cakes were extracted by following conventional extraction and ultrasound extraction technique following the protocol of Chen et al. (2012) along with the required modifications. For this purpose, solvents used were ethanol and distilled water at constant temperature (60°C), and at constant time (1.5 h for CE and 10 min for USE). Conventional extraction (CE) was done by utilizing an orbital shaker (Model 780, thermos fisher, US), while the ultrasound extraction (USE) was done by utilizing ultrasonic equipment (VCX 750, Newtown). Thereafter, extraction was performed by applying conventional and sonication techniques, and filtration of the mixture was done by using Whatman® filter paper (Whatman, UK). As a result, the final extracts of mustard oilseed cake (MOC) and sesame oilseed cake (SOC) were generated. The MOC and SOC extracts were then concentrated and finally changed into a powdered form by utilizing freeze‐drying. These were then stored at the optimum required conditions. The following processing conditions of time and temperature were chosen based on the former results of the polyphenol extraction by utilizing these modules followed by the same research group. In addition to this, in the preliminary experiments, these conditions revealed better consequences among the various tested processing combinations.

### 2.6 Phytochemical screening assays

The antioxidant assays were carried out through the TPC, DPPH, and FRAP assay estimation.

#### 2.6.1 Total phenolic contents (TPC)

Total phenolic contents were the most important assay to estimate the antioxidant potential of the tested MOC and SOC samples. For this purpose, an equal amount of FC reagent and the sample (MOC, SOC) was taken, along with 500 μl of distilled water. These were kept for 5 min.

Then, 4.5 ml of 7% Na2CO3 was added. After this, the mixture was allowed to stay for 90 min. Finally, the absorbance was calculated using a spectrophotometer (IRMECO, U2020) at 760 nm. The TPC was calculated as gallic acid equivalents (mg gallic acid/g) adapting the procedures of Hussain et al. (2019).

#### 2.6.2 Antioxidant potential

##### DPPH radical scavenging assay

DPPH (1,1‐diphenyl‐2‐picrylhydrazyl) free radical scavenging ability estimation is commonly used to determine the antioxidant potential of the tested compound. Briefly, the sample (MOC, SOC) and DPPH solution (0.12 mM) were added to the test tube in the ratio of 4 ml and 1 ml, respectively. Then, they were kept for 30 min in a dark place. The absorbance was calculated at 520 and recorded by utilizing a UV/visible spectrophotometer alongside the control and the blank (Ostadalova et al., 2011).

##### Ferric‐reducing antioxidant power (FRAP)

The metal ion chelation ability is another parameter to assess the antioxidant capacity of the tested compounds under different challenges and for this, ferric‐reducing antioxidant power is the most adopted test. For the estimation of FRAP, 0.5 ml of sample was added in 125 ml of phosphate buffer prepared 0.2 M and pH of 6.6 and potassium ferricyanide solution (1%) and placed in the water bath at 50°C for 15 min. In the resulting sample, an equal amount of 1.25 ml trichloroacetic acid (10%) and distilled water alongside 0.25 ml of ferric chloride (1%) was added kept for 10 min and the reading was recorded at 700 nm.

### 2.7 Statistical analysis

To check the level of significance, data for all parameters were statistically analyzed. One‐way and two‐way ANOVA were performed for analysis of variance and estimation of the significance among mean values. Tukey's HSD test was applied. The calculated values are represented as mean ± SD.

## 3 RESULTS AND DISCUSSIONS

### 3.1 Proximate analysis of mustard and sesame oilseed meals

Proximate composition is an essential factor for the assessment of raw material quality. Mustard oilseed cake was subjected to various quality trait assessments and showed moisture, carbohydrate, crude fat, crude protein, crude ash, and crude fiber as 1.8 ± 0.4, 21.16 ± 1.5, 21.10 ± 1%, 39.60 ± 0.8%, 7.6 ± 0.3%, and 8.2 ± 0.6, respectively (Table 2). The results are in consonance with the findings of Kuo, LH. (1967) that described moisture, carbohydrate, crude fat, crude protein, crude fiber, and crude ash at 1.5%, 19.5%, 20.1%, 38.5%, 3.5%, and 9.9%, respectively. Later, Sehwag and Das (2015) narrated the proximate composition of mustard cake results for moisture, carbohydrate, crude fat, crude protein, crude ash, and crude fiber at 1.5%, 21%, 21%, 33%–40%, 8.5%, and 8%, respectively. The variations in the current study may have occurred due to various differences, including climate and proximate methods.

**TABLE 2:** Compositional analysis of MOC and SOC

Proximate MOC SOC			Mineral%	MOC	SOC
Moisture	1.8 ± 0.4	7.4 ± 0.2	Ca	0.04	2.43
Carbohydrate	21.16 ± 1.5	28.0 ± 2.0	K	1.13	0.51
Crude Fat	21.10 ± 1%	27.5 ± 1.0	Mg	0.05	0.027
Crude Protein	39.60 ± 0.8%	31.0 ± 1.5	Na	0.06	0.0045
Crude Ash	7.6 ± 0.3%	5.6 ± 0.3	Cu	0.001	ND
Fiber	8.2 ± 0.6	0.8 ± 0.01	P	ND	1.11

ND Non detective.

Value indicate as mean with ±standard deviation.

Proximate analysis of sesame oil seed cake showed moisture contents, protein, fiber, fats, carbohydrate, ash, and contents as 7.4 ± 0.2, 28.0 ± 2.0, 27.5 ± 1.0, 31.0 ± 1.5, 5.6 ± 0.3, and 0.8 ± 0.01 (Table 2), respectively. The results of the current investigation regarding the proximate composition of sesame oilseed cake are in line with the earlier findings of Zebib et al. (2015), Hassan et al. (2012) when they carried out the proximate profile of sesame cake and observed moisture, protein, fat, ash, and fibers was moisture contents, fat, ash, protein, fiber, and carbohydrate% as 6.79 ± 0.36, 49.55 ± 0.48, 2.37 ± 0.02, 21.81 ± 0.53, 8.52 ± 0.13, and 10.94 ± 1.18, which are mean ± S.D, respectively. Later, Kuo (1967) carried out the proximate composition of peanut sesame cake and observed that moisture, ash, crude fiber, crude fat, crude protein, and carbohydrates were 6.01, 6.53, 7.6, 28.8, 35.6, and 11.01, respectively. The variations in the current study may have occurred due to various differences, including climate and proximate methods.

### 3.2 Mineral profile of mustard and sesame oilseed meal

Table 2 comprises calcium, potassium, magnesium, sodium, and copper as 0.04, 1.13, 0.05, 0.06, and 0.001, respectively. Sumitra et al. (2007) detected these minerals in mustard oilseed cake. The results for minerals such as calcium, phosphorus, potassium, magnesium, sodium, and copper were 0.05, 1.11, 1.12, 0.06, 0.05, and 0.01, respectively. Similarly, Kuo (1967) observed the mineral profile of mustard oilseed meal and described calcium, phosphorus, potassium, magnesium, sodium, and copper as 0.04, 0.98, 1.08, 1.15, 0.07, 0.04, and 0.02, respectively. The variations in the current study may have occurred due to various differences, including climate and methods.

### 3.3 Extraction and assessment of bioactive moieties

The mean square regarding the antioxidant indices of mustard and sesame oilseed meal illustrates the significant impact of each technique, solvent, and time interval on the traits.

However, their interactions showed a non‐significant trend.

### 3.4 Result & discussion

The means showed the highest polyphenols yield in ultrasound followed by conventional extraction. However, maximum recovery was obtained by aqueous ethanol compared to distilled water. Similarly, the highest ratio achieved in time for ultrasonic and less for conventional was 10 and 60 min followed by 15 and 90 min and minimum for 5 and 30 min, respectively (Table 3). Means exposed the highest MOC polyphenols extraction rate from ultrasonic while the lowest for the conventional technique was 5.64 ± 0.12 g GAE/100 g of mustard oilseed cake and 5.33 ± 0.13 g GAE/100 g of MOC, respectively. Among the solvents, ethanol caused a more potent effect as 5.79 ± 0.13 and 5.47 ± 0.15 g GAE/100 g of MOC followed by distilled water 5.50 ± 0.10 and 5.19 ± 0.12 g GAE/100 g of MOC in ultrasound and conventional extraction, respectively (Table 3). Our results showed that MOC has an appreciable amount of polyphenols. According to Ahmed et al. (2011), poly‐phenols yield is affected by various factors like extraction techniques and medium. Albu et al. (2004) reported that TPC extraction from MOC can be collected at a higher concentration by using ultrasound technology. Means exposed the highest sesame oilseed cake/SOC polyphenols extraction rate from ultrasonic, while the lowest for the conventional technique was 6.60 ± 0.01 g GAE/100 g of sesame cake and 6.29 ± 0.03 g GAE/100 g of SOC, respectively. Among the solvent, ethanol caused a more potent effect as 6.77 ± 0.03 and 6.45 ± 0.03 g GAE/100 g of SOC followed by distilled water 6.43 ± 0.02 and 6.13 ± 0.02 g GAE/100 g of SOC in ultrasound and conventional extraction, respectively (Table 3). The results of the current study are encouraging and are in line in the conclusion of Mohdaly et al. (2013, 2013) and Sarkis et al. (2014) as they described that polyphenols extraction obtained the optimum extraction with different solvents and time may also affect the extraction concerning TPC. Means regarding DPPH‐free radical scavenging activity also showed the same trend with high 64.26 ± 1.9 g TE/100 g MOC in UE and CE 63 ± 1.5 TE/100 g MOC for aqueous ethanol followed by water with 61.04 ± 1.5 and 59.8 ± 0.3 g TE/100 g MOC, respectively. Influence of time variations on extraction techniques explored the better activity 65.63 ± 1.4 and 64.35 ± 0.2 g TE/100 g MOC in UE at 10 min and C.E at 60 min than 62.65 ± 1.3 g TE/100 g MOC at 15 min and 59.90 ± 0.7 g TE/100 g MOC 90 min and lower 59.67 ± 0.7 g TE/100 g MOC g/100 g at 5 min and 58.50 ± 0.6gTE/100 g MOC 30 min, respectively. (Table 4) Dubie et al. (2013) extracted valuable antioxidants utilizing the ultrasound technique from MOC and found better results in UE than in the conventional method. Rubalya et al. (2015) also studied DPPH scavenging activity of mustard.

**TABLE 3:** Effect of treatments on total phenols content (g GAE/100 g of MOC & SOC)

Mustard oilseed cake									Sesame oilseed cake								
CE UE									CE UE								
Solvent 30 min 60 min 90 min Mean					5 min 10 min 15 min Mean				Solvent 30 min 60 min 90 min Mean 5 min 10 min 15 min Mean								
Ethanol	5.21 ± 0.01	5.73 ± 0.02	5.47 ± 0.09	5.47 ± 0.15	5.52 ± 0.01	6.07 ± 0.03	5.79 ± 0.02	5.79 ± 0.13	Ethanol	6.15 ± 0.03	6.76 ± 0.02	6.45 ± 0.04	6.45 ± 0.03	6.47 ± 0.01	7.09 ± 0.04	6.77 ± 0.03	6.77 ± 0.03
Water	4.94 ± 0.05	5.44 ± 0.1	5.19 ± 0.05	5.19 ± 0.12	5.23 ± 0.09	5.76 ± 0.2	5.59 ± 0.05	5.50 ± 0.10	Water	5.84 ± 0.01	6.42 ± 0.03	6.13 ± 0.02	6.13 ± 0.02	6.13 ± 0.02	6.74 ± 0.01	6.43 ± 0.02	6.43 ± 0.02
Mean	5.07 ± 0.09	5.50 ± 0.07	5.33 ± 0.05	5.33 ± 0.13	5.37 ± 0.05	5.92 ± 0.09	5.64 ± 0.07	5.64 ± 0.12	Mean	5.99 ± 0.02	6.59 ± 0.04	6.29 ± 0.03	6.29 ± 0.03	6.29 ± 0.03	6.91 ± 0.01	6.60 ± 0.02	6.60 ± 0.01

**TABLE 4:** Effect of treatments on DPPH test (g TE/100 g of MOC & SOC)

Mustard oilseed cake sesame oilseed cake																	
CE UE									CE UE								
Solvent 30 min 60 min 90 min Mean 5 min 10 min 15 min Mean									Solvent 30 min 60 min 90 min Mean 5 min 10 min 15 min Mean								
Ethanol	60 ± 0.3	66 ± 1.7	63 ± 0.9	63 ± 1.5	61.2 ± 0.9	67.3 ± 1.9	64.2 ± 1.2	64.26 ± 1.9	Ethanol	64 ± 0.6	70.4 ± 0.8	67.2 ± 0.6	67.2 ± 0.5	66.09 ± 2.4	72.68 ± 1.9	69.33 ± 2.1	69.37 ± 3.5
Water	57 ± 0.5	62.7 ± 0.8	59.85 ± 0.6	59.85 ± 0.3	58.14 ± 0.5	63.95 ± 1.5	61.04 ± 1.1	61.04 ± 1.5	Water	60.8 ± 0.4	66.8 ± 0.4	63.8 ± 0.4	63.8 ± 0.6	62.79 ± 1.8	69.06 ± 0.3	65.92 ± 0.6	65.92 ± 0.7
Mean	58.5 ± 0.6	64.35 ± 0.2	59.9 ± 0.7	61.42 ± 0.8	59.67 ± 0.7	65.63 ± 1.4	62.65 ± 1.3	62.65 ± 1.7	Mean	62.4 ± 0.2	68.6 ± 0.5	65.5 ± 0.2	65.5 ± 0.2	64.44 ± 2.2	70.87 ± 0.5	67.62 ± 0.9	67.64 ± 2.5

Means regarding DPPH‐free radical scavenging activity also showed the same trend with high 69.37 ± 3.5 g TE/100 g SOC in U.E and C.E 67.2 ± 0.5 TE/100 g SOC for aqueous ethanol followed by water with 65.92 ± 0.7 and 63.8 ± 0.6 g TE/100 g SOC, respectively. Influence of time variations on extraction techniques explored the better activity 70.87 ± 0.5 and 68.6 ± 0.5 g TE/100 g MOC in U.E at 10 min and C.E at 60 min than 67.62 ± 0.9 g TE/100 g MOC at 15 min and 65.50 ± 0.2 g TE/100 g MOC 90 min and lower 64.44 ± 2.2 g TE/100 g MOC g/100 g at 5 min and 62.4 ± 0.2 g TE/100 g MOC 30 min, respectively. (Table 3) The results of the present investigation are strengthened by the DPPH activity of SOC observed and a better performance of ultrasound technique could be noticed in their extract by Chang et al. (2002). Furthermore, parallel results were observed by Suja et al. (2004). Means of FRAP chelating metal ions activity were affected by the extraction techniques and solvents. Ultrasonic extraction technique exhibited 2.64 ± 0.02 g TE/100 g MOC maximum FRAP activity in contrast with the conventional extraction technique of 2.54 ± 0.02 g TE/100 g MOC, respectively. While the maximum chelating activity while solvent extraction was represented by aqueous ethanol using ultrasound and conventional as 2.71 ± 0.02 g TE/100 g MOC and 2.61 ± 0.02 g TE/100 g MOC, respectively. While in the case of water solvents with ultra‐sonic and conventional technique, the values were 2.57 ± 0.01 and 2.48 ± 0.01 g TE/100 g MOC, respectively. The current results regarding the antioxidant activity are compared with previous findings of Dubie et al. (2013); they selected high‐intensity ultrasound to extract the antioxidant or ferric‐reducing antioxidant potential of mustard. The means of FRAP chelating metal ions activity were affected by the extraction techniques and solvents. Ultrasonic extraction technique exhibited 3.31 ± 0.03 g TE/100 g SOC maximum FRAP activity in contrast with the conventional extraction technique which produced values of 3.19 ± 0.02 g TE/100 g SOC, respectively. The maximal chelating activity during solvent extraction represented by aqueous ethanol using ultrasound and conventional was observed as 3.40 ± 0.03 g TE/100 g SOC and 3.27 ± 0.02 g TE/100 g MOC, respectively. In the case of water solvents, with ultrasonic and conventional methods, results were observed as 3.23 ± 0.02 and 3.11 ± 0.01 g TE/100 g SOC, respectively (Table 5). Mohdaly et al. (2013, 2013) carried out the FRAP assay of sesame oilseed meal by using different solvents. Esmaeilzadeh Kenari et al. (2014) described that the results of ultrasound showed a maximum yield. Effects of time variations for both extraction methods gave us the conclusion that the mean of MOC extract through ABTS was 2.70 ± 0.03gTE/100 MOC and 3.47 ± 0.02gTE/100 g SOC by using ultrasound. The recorded result for the mean of ABTS in conventional was 2.62 ± 0.03 MOC and 3.34 ± 0.03 SOC (Table 6).

**TABLE 5:** Effect of treatments on FRAP test (g TE/100 g MOC & SOC)

Mustard oilseed cake sesame oilseed cake																	
CE UE									CE UE								
Solvent 30 min 60 min 90 min Mean 5 min 10 min 15 min Mean									Solvent 30 min 60 min 90 min Mean 5 min 10 min 15 min Mean								
Ethanol	2.49 ± 0.01	2.73 ± 0.02	2.61 ± 0.02	2.61 ± 0.02	2.58 ± 0.02	2.83 ± 0.02	2.71 ± 0.01	2.71 ± 0.02	Ethanol	3.12 ± 0.02	3.43 ± 0.03	3.27 ± 0.02	3.27 ± 0.02	3.24 ± 0.02	3.56 ± 0.03	3.40 ± 0.03	3.40 ± 0.03
Water	2.36 ± 0.01	2.60 ± 0.01	2.48 ± 0.01	2.48 ± 0.01	2.45 ± 0.01	2.70 ± 0.01	2.57 ± 0.02	2.57 ± 0.01	Water	2.96 ± 0.01	3.26 ± 0.01	3.11 ± 0.02	3.11 ± 0.01	3.07 ± 0.01	3.39 ± 0.02	3.23 ± 0.02	3.23 ± 0.02
Mean	2.42 ± 0.01	2.66 ± 0.01	2.54 ± 0.02	2.54 ± 0.02	2.52 ± 0.02	2.77 ± 0.02	2.64 ± 0.01	2.64 ± 0.02	Mean	3.04 ± 0.01	3.34 ± 0.03	3.19 ± 0.02	3.19 ± 0.02	3.16 ± 0.01	3.47 ± 0.02	3.31 ± 0.03	3.31 ± 0.03

**TABLE 6:** Effect of treatments on ABTS test (g TE/100 g MOC & SOC)

Mustard oilseed cake sesame oilseed cake																	
CE UE									CE UE								
Solvent 30 min 60 min 90 min Mean 5 min 10 min 15 min Mean									Solvent 30 min 60 min 90 min Mean 5 min 10 min 15 min Mean								
Ethanol	2.45 ± 0.02	2.69 ± 0.04	2.57 ± 0.03	2.57 ± 0.03	2.52 ± 0.02	2.77 ± 0.03	2.64 ± 0.02	2.64 ± 0.03	Ethanol	3.12 ± 0.02	3.43 ± 0.03	3.27 ± 0.02	3.27 ± 0.02	3.24 ± 0.02	3.56 ± 0.03	3.40 ± 0.03	3.40 ± 0.03
Water	2.32 ± 0.01	2.56 ± 0.03	2.44 ± 0.02	2.44 ± 0.01	2.38 ± 0.01	2.63 ± 0.03	2.51 ± 0.02	2.51 ± 0.01	Water	2.96 ± 0.01	3.26 ± 0.01	3.11 ± 0.02	3.11 ± 0.01	3.07 ± 0.01	3.39 ± 0.02	3.23 ± 0.02	3.23 ± 0.02
Mean	2.38 ± 0.01	2.62 ± 0.03	2.50 ± 0.02	2.50 ± 0.02	2.45 ± 0.01	2.70 ± 0.03	2.58 ± 0.02	2.58 ± 0.02	Mean	3.04 ± 0.01	3.34 ± 0.03	3.19 ± 0.02	3.19 ± 0.02	3.16 ± 0.01	3.47 ± 0.02	3.31 ± 0.03	3.31 ± 0.03

## 4 CONCLUSION

From the above findings, it has been concluded that mustard and sesame oilseed cake/meals have an excellent profile along with showing appreciable antioxidant potential. Among the extraction techniques, the ultrasound exhibited better extraction as well as isolation potential compared to conventional techniques. In case of plant material, ultrasound waves modify cavitation effect as well as physiochemical properties to stimulate the extractable compounds' release. Similarly, among solvents, ethanol proved to be more effective in comparison with distilled water. In MOC and SOC, the most abundant phenolic compounds (hydroxybenzoic acids) and (hydroxycinnamic acids) were found. MOC and SOC extracts can be utilized in the preparation of functional food products as a source of bioactive constituents. Further investigation on the incorporation of tested MOC and SOC extracts in various functional drinks is under process in our laboratory.

## FUNDING INFORMATION

No funding was received for this study.

## CONFLICT OF INTEREST

The authors declare no conflict of interest.

## ETHICAL APPROVAL

This article does not involve humans or animals.

## CONSENT TO PARTICIPATE

All the co‐authors are willing to participate in this study.

## CONSENT FOR PUBLICATION

All authors are willing for the publication of this manuscript.

## Data Availability

Even though adequate data have been given, all authors declare that if more data are required, then the data will be provided on a request basis.

## References

[fsn33214-bib-0001] AACC . (2000). Approved methods of American Association of Cereal Chemists. The American Association of Cereal Chemists.

[fsn33214-bib-0002] Abiodun, O. A. (2017). The role of oilseed crops in human diet and industrial use. In Oilseed crops: yield and adaptations under environmental stress (pp. 249–263). Willey.

[fsn33214-bib-0003] Ahmed, M. , Akter, M. S. , & Eun, J. B. (2011). Optimization conditions for anthocyanin and phenolic content extraction form purple sweet potato using response surface methodology. International Journal of Food Sciences and Nutrition, 62(1), 91–96. 20858156 10.3109/09637486.2010.511167

[fsn33214-bib-0004] Albu, S. , Joyce, E. , Paniwnyk, L. , Lorimer, J. P. , & Mason, T. J. (2004). Potential for the use of ultrasound in the extraction of antioxidants from Rosmarinus officinalis for the food and pharmaceutical industry. Ultrasonicssonochemistry, 11(3–4), 261–265. 10.1016/j.ultsonch.2004.01.015 15081992

[fsn33214-bib-0006] Cecilia, J. A. , García‐Sancho, C. , Maireles‐Torres, P. J. , & Luque, R. (2019). Industrial food waste valorization: A general overview. In J. R. Bastidas‐Oyanedel & J. Schmidt (Eds.), Biorefinery (pp. 253–277). Springer.

[fsn33214-bib-0007] Chang, L. W. , Yen, W. J. , Huang, S. C. , & Duh, P. D. (2002). Antioxidant activity of sesame coat. Food chemistry, 78(3), 347–354.

[fsn33214-bib-0008] Chen, C. , Chaudhary, A. , & Mathys, A. (2020). Nutritional and environmental losses embedded in global food waste. Resources, Conservation and Recycling, 160, 104912.

[fsn33214-bib-0009] Chen, H. J. , Inbaraj, B. S. , & Chen, B. H. (2012). Determination of phenolic acids and flavonoids in TaraxacumformosanumKitam by liquid chromatography–tandem mass spectrometry coupled with a post‐column derivatization technique. International Journal of Molecular Sciences, 13(1), 260–285. 22312251 10.3390/ijms13010260 PMC3269685

[fsn33214-bib-0011] Dubie, J. , Stancik, A. , Morra, M. , & Nindo, C. (2013). Antioxidant extraction from mustard (Brassica juncea) seed meal using high‐intensity ultrasound. Journal of Food Science, 78(4), E542–E548. 23488824 10.1111/1750-3841.12085

[fsn33214-bib-0201] Esmaeilzadeh Kenari, R. , Mohsenzadeh, F. , & Amiri, Z. R. (2014). Antioxidant activity and total phenolic compounds of Dezful sesame cake extracts obtained by classical and ultrasound‐assisted extraction methods. Food Science & Nutrition, 2(4), 426–435. 25473500 10.1002/fsn3.118 PMC4221841

[fsn33214-bib-0013] Hassan, A. A. , Rasmy, N. M. , Foda, M. I. , & Bahgaat, W. K. (2012). Production of functional biscuits for lowering blood lipids. World Journal of Dairy & Food Sciences, 7(1), 1–20.

[fsn33214-bib-0014] Hussain, G. , Saeed, F. , Shahbaz, M. , Ahmed, A. , Imran, M. , Khan, M. A. , Faiz, F. , Bano, Y. , Munir, R. , Nadeem, M. , & Jabeen, F. (2019). Reconnoitring the impact of different extraction techniques on ginger bioactive moieties extraction, antioxidant characterization and physicochemical properties for their therapeutic effect. Pakistan Journal of Pharmaceutical Sciences, 32(5), 2223–2236. 31894048

[fsn33214-bib-0015] Kaneria, M. J. , Bapodara, M. B. , & Chanda, S. V. (2012). Effect of extraction techniques and solvents on antioxidant activity of pomegranate (*Punicagranatum* L.) leaf and stem. Food Analytical Methods, 5(3), 396–404.

[fsn33214-bib-0016] Kaur, M. , Soodan, R. K. , Katnoria, J. K. , Bhardwaj, R. , Pakade, Y. B. , & Nagpal, A. K. (2014). Analysis of physico‐chemical parameters, genotoxicity and oxidative stress inducing potential of soils of some agricultural fields under rice cultivation. Tropical Plant Research, 1(3), 49–61.

[fsn33214-bib-0017] Kumar, C. M. , & Singh, S. A. (2015). Bioactive lignans from sesame (*Sesamumindicum* L.): Evaluation of their antioxidant and antibacterial effects for food applications. Journal of Food Science and Technology, 52(5), 2934–2941. 25892793 10.1007/s13197-014-1334-6 PMC4397349

[fsn33214-bib-0018] Kuo, L. H. (1967). Animal feeding stuffs compositional data of feeds and concentrates (part 3). The Malaysian Agricultural Journal, 46(1), 63–70.

[fsn33214-bib-0020] Mohdaly, A. A. A. , Hassanien, M. F. R. , Mahmoud, A. , Sarhan, M. A. , & Smetanska, I. (2013). Phenolics extracted from potato, sugar beet, and sesame processing by‐products. International Journal of Food Properties, 16(5), 1148–1168.

[fsn33214-bib-0021] Ostadalova, M. , Pazout, V. , Straka, I. , Tauferova, A. , Bartl, P. , & Pokorna, J. (2011). Monitoring tea pigments theaflavins and Thearubigins in dependence on the method and duration of storage. Journal of Food Technology, 9(2), 50–56.

[fsn33214-bib-0022] Ramachandran, S. , Singh, S. K. , Larroche, C. , Soccol, C. R. , & Pandey, A. (2007). Oil cakes and their biotechnological applications–A review. Bioresource technology, 98(10), 2000–2009. 17023161 10.1016/j.biortech.2006.08.002

[fsn33214-bib-0023] Rodriguez‐Saona, L. E. , & Wrolstad, R. E. (2001). Extraction, isolation, and purification of anthocyanins. Anthocyanins. Current Protocols in Food Analytical Chemistry, (1), F1–1.

[fsn33214-bib-0200] Rubalya, V. S. , Mukesh Kumar, V. , & Devasena, T. (2008). Modeling temperature dependent viscosity parameter of vegetable oil and study of physicochemical properties on heating. Journal of Food Science and Technology, 53(2), 1328–1337. 10.1007/s13197-015-2050-6

[fsn33214-bib-0024] Rusak, G. , Komes, D. , Likić, S. , Horžić, D. , & Kovač, M. (2008). Phenolic content and antioxidative capacity of green and white tea extracts depending on extraction conditions and the solvent used. Food Chemistry, 110(4), 852–858. 26047270 10.1016/j.foodchem.2008.02.072

[fsn33214-bib-0025] Sadh, P. K. , Duhan, S. , & Duhan, J. S. (2018). Agro‐industrial wastes and their utilization using solid state fermentation: A review. Bioresources and Bioprocessing, 5(1), 1–15.

[fsn33214-bib-0026] Sarkis, J. R. , Michel, I. , Tessaro, I. C. , & Marczak, L. D. F. (2014). Optimization of phenolics extraction from sesame seed cake. Separation and Purification Technology, 122, 506–514.

[fsn33214-bib-0027] Sehwag, S. , & Das, M. (2015). A brief overview: Present status on utilization of mustard oil and cake.

[fsn33214-bib-0028] Shahidi, F. , Wanasundara, U. N. , & Amarowicz, R. (1994). Natural antioxidants from lowpungency mustard flour. Food Research International, 27(5), 489–493.

[fsn33214-bib-0029] Suja, K. P. , Abraham, J. T. , Thamizh, S. N. , Jayalekshmy, A. , & Arumughan, C. (2004). Antioxidant efficacy of sesame cake extract in vegetable oil protection. Food chemistry, 84(3), 393–400.

[fsn33214-bib-0100] Sumitra, R. , Sudheer, K. S. , Christian, L. , Carlos, R. S. , & Ashok, P. (2007). Oil cakes and their biotechnological applications ‐ A review. Bioresource Technology, 98, 2000–2009. 17023161 10.1016/j.biortech.2006.08.002

[fsn33214-bib-0030] Sultana, B. , Anwar, F. , & Ashraf, M. (2009). Effect of extraction solvent/technique on the antioxidant activity of selected medicinal plant extracts. Molecules, 14(6), 2167–2180. 19553890 10.3390/molecules14062167 PMC6254218

[fsn33214-bib-0033] Zebib, H. , Bultosa, G. , & Abera, S. (2015). Physico‐chemical Properties of Sesame (*Sesamumindicum indicum* L.) varieties grown in northern area, Ethiopia. Agricultural Sciences, 6(2), 238–246.

